# Infections and Multiple Sclerosis: From the World to Sardinia, From Sardinia to the World

**DOI:** 10.3389/fimmu.2021.728677

**Published:** 2021-10-06

**Authors:** Jessica Frau, Giancarlo Coghe, Lorena Lorefice, Giuseppe Fenu, Eleonora Cocco

**Affiliations:** Multiple Sclerosis Centre, Azienda Tutela Salute (ATS) Sardegna, University of Cagliari, Cagliari, Italy

**Keywords:** multiple sclerosis, Epstein - Barr virus, human endogeneous retrovirus-W, mycobacterium avium subspecies paratuberculosis, infections, genetic, Sardinia (Italy)

## Abstract

Multiple Sclerosis (MS) is an inflammatory disease of the central nervous system. Sardinia, an Italian island, is one of the areas with the highest global prevalence of MS. Genetic factors have been widely explored to explain this greater prevalence among some populations; the genetic makeup of the Sardinians appears to make them more likely to develop autoimmune diseases. A strong association between MS and some infections have been reported globally. The most robust evidence indicating the role of infections is MS development concerns the Epstein-Barr virus (EBV). Anti-EBV antibodies in patients once infected by EBV are associated with the development of MS years later. These features have also been noted in Sardinian patients with MS. Many groups have found an increased expression of the Human endogenous retroviruses (HERV) family in patients with MS. A role in pathogenesis, prognosis, and prediction of treatment response has been proposed for HERV. A European multi-centre study has shown that their presence was variable among populations, ranging from 59% to 100% of patients, with higher HERV expression noted in Sardinian patients with MS. The *mycobacterium avium subspecies paratuberculosis* (MAP) DNA and antibodies against MAP2694 protein were found to be associated with MS in Sardinian patients. More recently, this association has also been reported in Japanese patients with MS. In this study, we analysed the role of infectious factors in Sardinian patients with MS and compared it with the findings reported in other populations.

## Introduction

Multiple Sclerosis (MS) is an autoimmune disease of the central nervous system with an increasing incidence and prevalence in geographical regions with higher latitude, in both the hemispheres (1). Recent data indicates the incidence of MS in areas of lower latitude, as well as its prevalence, has increased in Europe and North America, with a high MS incidence in some southern areas (2). Nevertheless, it is known as an exception to this gradient Sardinia, an Italian island where the presence of MS is among the highest worldwide. The prevalence of the disease in Sardinia is 361/100,000 inhabitants (3). The Sardinian population has a peculiar genetic background that could partly explain this primacy (4, 5). To note, the main predisposing risk factor for MS in North Europeans is the HLA-DRB1*15 (*DRB1-15:01-DQB1*06:02), while in Sardinian population the predisposing *15:01 is virtually absent and MS is associated in particular to *13:03-*03:01, *04:05-*03:01, and *03:01-*02:01 (4, 5). On the other hand, a protective role in Sardinians has been found for DRB1*15:02, DRB1*16:01, DQB1*06:01, and DQB1*05:02 (4). Thus, the propensity to MS in Sardinians may be due to a complex presence of various HLA-DRB1-DQB1, from which the modality of antigen presentation depends (5).

Moreover, it is known that genetic factors alone are not sufficient for the presentation of the disease, and many environmental factors, particularly that affect the individuals in the first decades of life, are involved in the pathogenesis of MS (2, 6). Typically, the risk factors of MS have been classified into two groups: non-infectious and infectious. In the first group, lack of sunlight exposure, low levels of vitamin D, cigarette smoking, and a diet high in animal/saturated fats are reportedly associated with the development of MS (2, 6).

The association between infectious factors and the development of MS has been widely reported for a long time. Even in the seventies, elevated titres of rubeola, measles, and vaccinia viruses were noted in the cerebrospinal fluid (CSF) of patients with MS, and a role of persistent or slow infections was hypothesised (7). Particular attention was given to herpes viruses, while in the eighties, an Italian cooperative study found higher titres of antibodies against herpes simplex 2 and Epstein Barr virus (EBV) in patients with MS than in healthy controls and those with other neurological diseases (8). A possible association with Herpes human virus 6 (HHV-6) has been hypothesised from the nineties; however, controversial results have been described (9). To note, a recent metanalysis confirmed an association between HHV-6 and MS (10). Antibodies against measles, rubella, and herpes zoster virus (MRZ reaction) are present in 80%–100% of patients with MS (11), and their presence in patients with clinically isolated syndrome appears to predict the conversion to MS (12). Data about varicella-zoster, herpes simplex 1 and 2, and cytomegalovirus are conflicting and inconclusive (13).

The viruses involved in MS could manipulate the gene expression of the host, leading to immune deregulation and tissue damage; moreover, they can enter the brain and establish latent chronic infection (14).

Different mechanisms have been hypothesised for the involvement of viruses in MS pathogenesis and they are not mutually exclusive. Viruses can act as triggers or co-factors in the development of the disease. They could cause direct toxicity in the affected neurons (9); the T cell receptor (TCR) in the T lymphocyte surface could be specific for both virus and myelin antigens (15); after causing tissue damage, viruses can facilitate an over-reactive inflammation due to the exposition of hidden antigens, with the production of autoreactive immunity cells (bystander activation) (16); secondary to myelin disruption, its fragments are released in the inflammatory environment and new epitopes are exposed to inflammatory cells (epitope spreading) (17). The control of autoreactive cells is dependent on T regulatory cells; however, they appear defective in MS (18).

The more accredited mechanism to explain the involvement of viruses in autoimmune diseases such as MS is molecular mimicry. T cells specific for non-self epitopes cross-react with self-epitopes that are similar peptides, causing tissue damage (19, 20). In the case of MS, molecular mimicry between myelin basic protein (MBP) and EB nuclear antigen 1 (EBNA-1) is well established, and peptides deriving from both MBP and EBNA-1 could activate CD8+ T cells isolated from patients with MS (20). Moreover, T helper 1 (Th1) and Th1/17 are the main subsets in MS responding to peptides derived from myelin and viruses as EBV. Th1/17 isolated from CSF of patients with MS react both with self-antigen-presenting cells and peptides from viruses and bacteria (21).

To note, many disease-modifying therapies target cells in which viruses such as EBV and Human endogenous retroviruses (HERV) have been found, and it has been postulated that part of their effects could be explained by reducing HBV and HERV-W autoreactive cells (13).

### EBV

EBV is related to several diseases that develop years after the primary infection. It has been hypothesised that a decreased capacity to control EBV by the immune system could promote the onset of late EBV-related diseases, such as cancer, MS, and other autoimmune disorders (22). A defective T cell control of EBV in patients with MS has been reported (23).

The prevalence of EBV seropositivity in patients with MS is approximately 100% and a history of mononucleosis is a risk factor in the development of the disease (24, 25). Various studies estimate that previous EBV infection with mononucleosis increases the risk of MS to the same degree as that of carrying human-leukocyte antigen (HLA) DRB1 15*01, which is the strongest genetic factor associated with the disease (26). The detection of anti-EBV antibodies is associated with the onset of MS after 5–20 years after EBV infection (27).

Signs of EBV infections in the brain and CSF of patients with MS have been reported by some researchers, whereas this was not reported in other studies (28). Nevertheless, the presence of EBV in the brain could be not a peculiar characteristic of MS. Moreover, many viruses persisting in a latent stage are neurotrophic and could be found inside the brain (22). Recently, antibodies against specific epitopes in EBNA-1 cross-reacting with MBP have been recognised in the sera of patients with MS and not of healthy controls (29).

If the detection of EBV antibodies in patients with MS is increased, similar results are not found searching for DNA in biological fluids like blood, CSF, and saliva. This datum suggests that the role of EBV is not related to reactivation, which could be occasionally possible, but to the latent infection (22).

Various mechanisms have been proposed to explain EBV involvement in MS pathogenesis, such as molecular mimicry with MBP, an autoimmune response against alpha-beta-crystallin, and both complement and antibody-dependent cellular cytotoxicity (13).

EBNA-2 inside the host cells binds within genetic loci associated with MS (30). By EBNA-2 binding site, EBV could convert resting B cells into immortal B cells, which maintain autoreactive cells within the brain and blood of patients with MS (31).

In the MS Sardinian population, EBV DNA was detected more in patients than in controls (32), and a higher prevalence of anti EBNA1 antibodies has been found in those without disease-modifying treatments (DMTs) when compared to healthy controls (33). Antibodies titres decreased after 6 months of interferon beta, both in the Sardinian cohort and in other populations (33, 34).

Regarding DMTs, it has been hypothesised that CD20 monoclonal antibodies could exert their efficacy in eliminating EBV-infected memory B cells (22). In addition, the effect of natalizumab could be partially related to reduced mobility of EBV into CNS, both inhibiting the entry of EBV mediated by integrin, and blocking the lymphocyte trafficking, including that of EBV-infected B cells and EBV-directed T cells (35). Additionally, teriflunomide could influence the immune response to EBV and reduce their lytic replication (36).

### HERV

They are a group of retroviruses incorporated in the human genome millions of years ago, representing approximately 8% of the entire genome, and have regulatory functions for human gene expression (13). In contrast, when their expression is inappropriate, they should cause inflammation, aberrant immune expression, and deregulated gene expression (37–39).

The association of MS with the three types of HERV (HERV-H, HERV-K, and HERV-W), in particular the latter, has been demonstrated by various studies (40). Nevertheless, the presence of HERV-W is not specific for MS and is also found in patients with other non-inflammatory neurological diseases (41), and in close relatives of patients with MS, possibly being a predisposing factor for the disease (42). In contrast, in a study analysing north-Sardinian population, the association was found with MS and myelin oligodendrocyte glycoprotein (MOG)-IgG associated disorders, but not with optic neuromyelitis (NMO) (43). There is a probably molecular mimicry between HERV-W antigen and oligodendrocytes, which are primarily affected in MS, but not with astrocytes, which are primarily affected in NMO.

The proportion of patients with MS positive for HERV-W MS-associated retrovirus (MSRV) varies from 50% to 100% among the populations (44–46). This variability could be explained both with the heterogeneity of populations in terms of HERV expression, and the different detection methods used (13). In a European multicentre study, HERV-W was detected in approximately 100% of Sardinian patients with MS, while its presence was lower in other populations. Probably, a genetic selection in an isolated island has promoted the genome of Sardinians to contain high copies of HERV-W, with better circulation in a closed population (46). The presence of HERV-W viraemia has also been detected in 9% of healthy Caucasian blood donors (47); its positivity in the blood of patients with initially isolated optic neuritis is higher than in healthy controls and predicts the conversion to MS in the next 20 months (48).

In general, its expression could be modified not only by mutations, but also by retroviral restriction factors, external events such as viral infections, and epigenetic mechanisms and could directly activate the retrovirus or deregulate the mechanism preventing its expression (40). To note, an altered epigenetic regulation has been described in patients with MS (49), and the same alteration could be involved in the expression of MSRV.

HERV-W envelope (env) protein has been detected in the surface of macrophages and microglia in the brain of patients with MS near demyelinating lesions, and this protein share homologous sequences with MOG, being able to activate T and B cells (50).

Studies performed in patients with MS from Sardinia found that the presence of HERV-W products in the blood has been associated with poor prognosis (48, 51), while in the CSF it is more detected during relapses than during the remission phase (41), and its presence in CSF is associated with disability accumulation and higher relapse rate (51).

The MSRV load is higher in women with active MS, and it is correlated to a higher Expanded Disability Status Scale (EDSS)/Multiple Sclerosis Severity Score (MSSS) (52). The expression of MSRV is higher in MS patients with secondary progressive courses (40).

Regarding the detection of antibodies against HERV-W and HERV-H, they are higher during relapses than in the remission phase, and their levels correlate with higher expression of HERV-W env and HERV-H env in B cells and monocytes only during relapses (53).

A study performed in a Sardinian population shown that treatment with natalizumab reduced the expression of the MSRV/syncytin-1/HERV-W (54), as well viremia fell below detection limits during efficacious therapy with interferon beta and other treatments as fingolimod, azathioprine, and glatiramer acetate (55, 56).

The main implicated proteins are MSRV env and syncytin-1. They are expressed by B cells, monocytes, natural killer but not T cells (57), and have pro-inflammatory and superantigenic properties, and could cause inflammation, neurodegeneration, stress response, and altered immune response (56).

It is known that HERV-W cross-react with MOG, and it can bind CD14 and Toll-like receptor 4, promoting the release of pro-inflammatory cytokines (13).

Furthermore, its expression in myeloid cells into CNS induces a degenerative phenotype with axonal damage, and could also inhibit remyelination by reduced formation of oligodendrocytes precursor cells and promotion of pro-inflammatory cytokines (58). This last effect could be blocked by an anti-HERV monoclonal antibody (59).

The retrovirus can be activated by infections, and EBV appears to be its main activator. In particular, it could be triggered by the latent EBV phase, acting as an effector for MS (57, 60). Their interaction in the blood could induct the HERV-W env superantigen properties, while in the brain toxic mechanisms against oligodendrocytes could be involved, enhancing inflammation, demyelination, and axonal damage (57). High titres of anti-EBNA antibodies can be observed 15–20 years before the MS onset (61), with high expression of HERV-W simultaneously (60).

### *Mycobacterium avium Subspecies Paratuberculosis* (MAP)

Different mycobacteria have been identified in association with MS, and the TCR levels, important in recognizing mycobacteria, are increased in patients with MS (62).

It was primarily associated with MS in a Sardinian population (63). In particular, MAP DNA has been repeatedly found in a higher percentage of patients with MS than in healthy donors (32, 63, 64). Moreover, MAP antigens sharing epitopes with MS-related proteins can promote a high immune response both in blood and CSF of patients with MS, probably acting by molecular mimicry (63–65). In particular, a humoral response against the MAP2694 antigen, which is homologous of TCR gamma chain C region and the complement component 1, was found in approximately 34% of patients and only 10% of healthy controls (64). Moreover, heat shock proteins (HSP) of mycobacteria share homologous sequences with human proteins, thus promoting an autoimmune response by molecular mimicry (66). It is known that the lymphocyte proliferative response against some HSPs derived from mycobacteria is higher in patients with MS than in those with other neurological diseases or healthy controls (67, 68). Sardinian patients with MS have increased levels of antibodies against the MAP recombinant protein FprB and the MAP HSP70 (32, 69). In contrast, no antibodies have been found against a MAP epitope homologous to MOG (70).

Several MAP proteins and antigenic epitopes can induce a stronger immune response in patients with MS than in other patients (70).

Moreover, antibodies against several homologous epitopes of MAP, EBV, and humans have been found in CSF and blood samples of patients with MS (65). A common target of MAP and EBV in triggering autoimmune response could be MBP. In particular, both the pathogens should induce antibodies that cross-recognise some MBP peptides, which have conformational similarities with MAP and EBV antigens (65, 71, 72).

In a small study performed in Sardinian patients, cross-recognition of MAP and EBV epitopes (BOLF1_305-320_ and MAP_4027_18-32_) and the self-epitope IRF5_424-434_ appears to regulate the immune dysregulation in MS (73). Later, another study confirmed that in patients with MS, autoantibodies recognising MBP and IRF5 cross-react with homologous peptides from MAP and EBV, primarily owing to molecular mimicry.

It is likely that similar to other autoimmune diseases, MAP infection could enhance the risk of MS; however, other factors such as genetic susceptibility are fundamental for the development of MS. In individuals with higher susceptibility to mycobacterial infections, gene polymorphisms related to MS, such as the HLA complex, vitamin D receptor, TCL, and others have been detected (74).

In the Sardinian cohort of patients with MS, a lower proportion of patients carrying oligoclonal bands were MAP positive, probably because the action of MAP was primarily expressed in the periphery (64). Moreover, MAP DNA was detected more commonly in patients with recent steroid pulse therapy, while anti MAP2694 antibodies were detected in those taking interferon beta (64, 75).

More recently, an association between MAP and MS was found in Japan. A higher response of antibodies against surface antigens has been detected, with antibodies against MAP2694 present in 30% of patients, a significantly higher rate compared to healthy controls and those with other neurological diseases (76, 77). It has been highlighted that this MAP antigen can bind more to the HLA 1501 and 0301, which predisposes to MS than the protective haplotypes 1601 and 15*02. Thus, T cells are easily activated for inducing autoimmunity (78). In Sardinian and Japanese patients with MS the disease is associated with DPB1*0301, which is a genetic peculiarity (77).

The association between genetic factors and MAP has been analysed in Sardinian patients, and no association has been found with MAP positivity and predisposing haplotypes. Nevertheless, there was a lower detection of anti MAP antibodies in patients carrying at least one protective haplotype *versus* those without it (79). Moreover, an association between MAP and SLC11A1 polymorphism has been found consistently in the Sardinian MS cohort (80). This gene is important in the defence against intracellular pathogens; however, its role as an MS-associated gene is controversial (80).

When Japanese patients with MS and NMO were compared, anti MAP2694 antibodies were higher in blood and CSF of the first group than that of the latter (81). In NMO Sardinians patients, a humoral response against MAP antigens has been detected; however, there was no relationship between this response and the presence of anti AQP4 antibodies. Moreover, the same study demonstrated that MAP is unable to infect and persist within astrocytes (82).

## Conclusions

Many infectious factors have been studied worldwide for their presumed association with MS, both in its pathogenesis and the course of the disease.

EBV is the primarily implicated, and MS could be retained, as some types of cancer, a rare and late complication of EBV infection.

HERV-W has been found to be associated with MS in many populations; however, the incidence varies with the geographical area. A strong dependence has been found from EBV infection in triggering MS.

MAP has been studied only in Sardinia and Japan and both the populations are associated with MS. As postulated by other authors the relationship between MAP and MS could be population-specific, and dependent on both genetic and non-genetic factors (77).

Sometimes, a genetic variant protective against a specific disease increases the risk of another disease. This is the case of a variant of tumour necrosis factor superfamily13B, gene encoding for the B-cell activating factor (BAFF). It was selected in the past in Sardinia as it could provide resistance from malaria, which was endemic on the island. In contrast, it enhances levels of BAFF, B cells, and immunoglobulins, favouring autoimmunity similar to MS (83).

Recently, the PRF1:p.A91V mutation, which causes an increase of lymphocytes (particularly cytotoxic memory T cells) and perforin deficiency, has been associated with MS. This same mutation, when reinforced by environmental factors such as herpes infections, could favour an exaggerated activation of the immune system and trigger familial haemophagocytic lymphohistiocytosis (84).

In conclusion, genetic and other environmental factors are critical for the onset of MS, which appears to be dependent on their complex interaction.

## Author Contributions

JF collected the literature, designed the review, and wrote the paper. GC designed, wrote and revised critically the paper. LL and GF designed and revised critically the paper. EC concepted, designed and revised critically the review. All authors contributed to the article and approved the submitted version.

## Conflict of Interest

The authors declare that the research was conducted in the absence of any commercial or financial relationships that could be construed as a potential conflict of interest.

## Publisher’s Note

All claims expressed in this article are solely those of the authors and do not necessarily represent those of their affiliated organizations, or those of the publisher, the editors and the reviewers. Any product that may be evaluated in this article, or claim that may be made by its manufacturer, is not guaranteed or endorsed by the publisher.
